# Hooray for Hypoxia?

**DOI:** 10.1371/journal.pmed.0020130

**Published:** 2005-06-28

**Authors:** Geoff Bellingan

## Abstract

The mortality of critically ill patients rises steadily as the partial pressure of arterial oxygen falls below about 11 kPa (80 mm Hg). A new animal study in the May 2005 issue of *PLoS Biology* showing a potential benefit for hypoxia is thus a challenge to current thinking.

## Teaser

A new study comes to a counter-intuitive conclusion: hypoxia may have its benefits for patients with lung injury

## The Risks of Hypoxia and Hyperoxia

**Figure 1:**
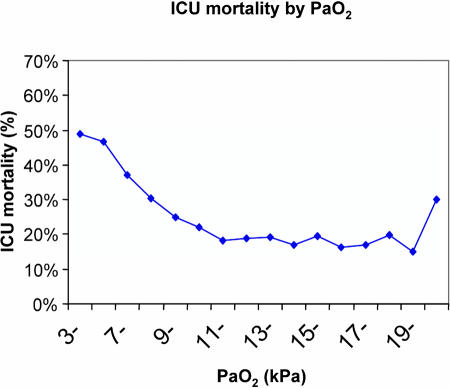
Mortality Rises Steadily as PaO_2_ Falls

Hypoxaemia kills. Recently we have shown, in a study of over 50 000 critically ill patients, that mortality rises steadily as the partial pressure of arterial oxygen (PaO_2_) falls below about 11 kPa (80 mm Hg) (Figure 1) [1]. Rats exposed to hypoxic conditions develop a mild inflammatory response with increased capillary leak and pro-inflammatory cytokine release [2]. Hypoxia increases leukocyte adhesion, prolongs neutrophil survival, enhances pro-inflammatory responses (in particular, increasing leukocyte IL-8 expression), and reduces lung fluid transport [3,4]. A new study by Thiel et al. in *PLoS Biology* that shows a potential benefit for hypoxia is therefore a challenge to current thinking [5].

To put this finding into context we must acknowledge the equally accepted fact that hyperoxia is toxic. Experiments in primates show that 100% oxygen results in progressive damage to the pulmonary endothelium and epithelium. It causes free radical release, capillary leak, and impaired surfactant function in addition to arteriolar vasoconstriction and maldistribution of microcirculatory perfusion [6,7].

What, then, is the clinical balance we should strike between these extremes? Oxygenation of patients with acutely impaired lung function saves lives, but what is the right amount of oxygen? Many clinicians use the oxygen dissociation curve, aiming for saturations of above 92%. Others highlight the fact that patients with severe respiratory failure can survive despite being mechanically ventilated for days on 100% oxygen. In other words, a high percentage of inspired oxygen itself may not be harmful. (In animal studies showing harm from giving 100% oxygen, the lungs are usually normal at the beginning of the study, so the arterial oxygen is correspondingly high—typically a PaO_2_ of 40 kPa (300 mm Hg); thus, it may be the high PaO_2_ rather than the high percentage of inspired oxygen that causes lung damage.) Some researchers question the importance of oxygen toxicity at all—for example, Meier et al., using a haemorrhagic shock model, found a significant improvement in outcome from the addition of 100% oxygen to the resuscitation [8].

## The New Study in Mice: Hyperoxia May Enhance Tissue Damage

**Figure 2:**
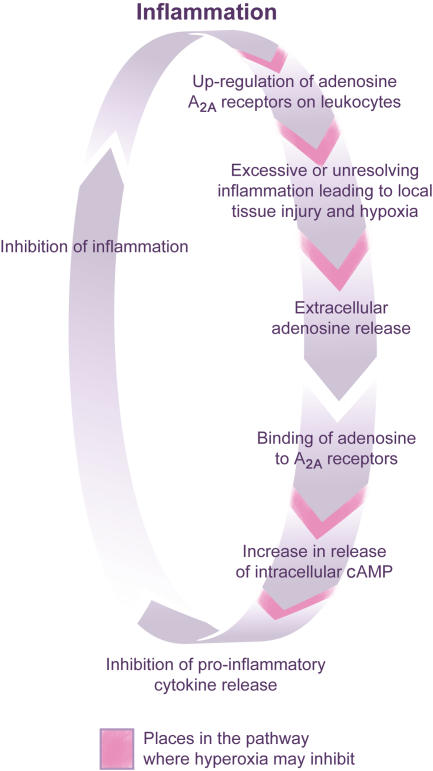
Adenosine Receptors Down-Regulate Inflammation through a Negative Feedback Loop Sitkovsky and colleagues postulate that inflammation causes tissue damage leading to adenosine release, which acts on adenosine A_2A_Rs to increase intracellular cyclic AMP. The rise in cyclic AMP down-regulates the inflammatory response. Tissue inflammation induces local hypoxia, which regulates the adenosine-driven protective response. The figure shows the steps in the pathway where hyperoxia might act (having an inhibitory effect). (Illustration: Giovanni Maki)

It is against this background that the study by Thiel et al. is so interesting. Two of the study's authors, Ohta and Sitkovsky, showed recently that adenosine receptors are important in down-regulating inflammation [9]. They argued that a negative feedback loop operates: inflammation causes tissue damage, leading to adenosine release, which acts on G-protein-coupled adenosine A_2A_ receptors (A_2A_Rs) to increase intracellular cyclic AMP, which in turn reduces the activity of NFκB and down-regulates the inflammatory response (Figure 2). Indeed, inflammation was dramatically enhanced in A_2A_R-deficient mice compared with wild-type controls. Importantly, no significant compensatory changes in the expression of other adenosine receptors have been demonstrated in these A_2A_R-deficient mice.

Sitkovsky and colleagues went on to propose that tissue inflammation induced local hypoxia and that this hypoxia was important in regulating the adenosine-driven protective response. As a corollary they postulated that hyperoxia interrupts the hypoxia→adenosine→A_2A_R lung protection pathway and hence could enhance tissue damage (Figure 2). The current paper by Sitovsky's group in *PLoS Biology* examines this hypothesis [5].

At the outset, using an inflammatory challenge that significantly impaired gas exchange, the authors confirmed that when mice were additionally exposed to 100% oxygen, the toxicity of the challenge was dramatically increased compared to the inflammatory challenge in normoxic conditions (21% oxygen). (Over the time frame of the experiments high concentrations of oxygen alone were found to be only mildly inflammatory.) Inflammatory neutrophils from the challenged mice expressed the A_2A_R, and exposure to high concentrations of oxygen reduced this expression. Moreover, an adenosine agonist could block pro-inflammatory responses.

Mice lacking the A_2A_R had increased neutrophil infiltration in the lungs after endotoxin challenge and poorer gas exchange. The same was seen in wild-type mice treated with an A_2A_R blocker. Endotoxin-challenged mice breathing 10% oxygen (hypoxia) had a low mortality and, in the survivors, lung inflammation was less severe than in mice breathing room air. Hypoxia also significantly reduced pulmonary neutrophil accumulation and improved gas exchange compared with normoxia, suggesting that hypoxia acted to protect the lung from additional inflammatory damage. Importantly, hypoxia was also associated with elevated adenosine concentrations.

In contrast to the 20% mortality of hypoxic, endotoxin-challenged wild-type mice, hypoxic, endotoxin-challenged A_2A_R-deficient mice had elevated local and systemic accumulation of pro-inflammatory cytokines, and all died. As hypoxia per se did not affect the A_2A_R-deficient mice, this suggests that both hypoxia and A_2A_R functionality are required to induce the anti-inflammatory response. Finally, the authors showed that an adenosine agonist also inhibited lung injury in endotoxin-treated mice, reducing leukocyte influx and capillary leak. Importantly, this agonist could dramatically improve mortality even with a severe two-hit inflammatory challenge under hyperoxic conditions.

## Implications of the Study

These are exciting and provocative results. They are, however, hard to reconcile with many studies showing pro-inflammatory effects from hypoxia. Sitkovsky and colleagues attempt to reconcile this difference by saying that their study had a long time course (over 48 hours), whereas studies showing that hypoxia is harmful usually had much shorter study periods.

The authors' explanation is probably too simplistic, and many issues and questions remain. (1) Different challenges—for example, pulmonary versus extrapulmonary—might give different results. (2) There is emerging evidence that more prolonged hypoxia can affect many processes, for example, mitochondrial function, and such complicated metabolic changes may be important and need to be examined [10]. (3) We don't know the mechanism of increased extracellular adenosine. (4) Lung injury may relate to tachypnoea and tidal volume, and these have not been measured. (5) In sampling arterial oxygen 15 minutes after removal from the hyperoxia, we do not know whether the animals were exposed to excessive PaO_2_ levels or whether the degree of lung injury prevented the animals from having excessive PaO_2_ levels. In other words, although the animals were given 100% oxygen, they may not have had an elevated PaO_2_ (the lung injury may have prevented oxygenation of the blood). This issue is critically important, as the use of high-percentage inspired oxygen simply to achieve a normal PaO_2_ is much more akin to clinical practice.

Refreshingly, though, this is a study that invites us to re-evaluate our perspectives. We know hypoxia can be bad, but is it as bad as hyperoxia? The scenario is all too familiar—the patient's lung injury has rendered the patient hypoxaemic, and the paramedics have started high-flow oxygen. In the emergency room the team are heartened to see the patient “pink up” and to hear the ominous sound of the saturation monitor abate. But, beware, is the team *really* in a comfort zone? Is the saturation monitor really now chirping happily with 100% oxygen saturations, or is this the harbinger of future problems as the PaO_2_ increases unnoticed? Should we have an additional set of alarm limits, hitherto unrecognised: those of high oxygen tensions? Should the monitors be alarming not only when arterial saturation is below 90% but also when it's above 98%? The study by Thiel et al. suggests that we urgently need to find out.

## Abbreviations

A2AR: adenosine A2A receptor
PaO2: partial pressure of arterial oxygen
